# Effects of harvesting and an invasive mussel on intertidal rocky shore communities based on historical and spatial comparisons

**DOI:** 10.1371/journal.pone.0294404

**Published:** 2024-02-08

**Authors:** Ndiviwe G. Baliwe, Maya C. Pfaff, George M. Branch

**Affiliations:** 1 Department of Biological Sciences and Marine Research Institute, University of Cape Town, Rondebosch, South Africa; 2 Cape Research Centre, SANParks Scientific Services, Cape Town, South Africa; 3 Oceans and Coastal Research, Department of Forestry, Fisheries and Environment, Cape Town, South Africa; UMinho CBMA: Universidade do Minho Centro de Biologia Molecular e Ambiental, PORTUGAL

## Abstract

Intertidal rocky shores are the most accessible marine habitats and therefore heavily impacted by harvesting. In recent years, they have also been increasingly invaded by alien species, which compounds the effects of harvesting on rocky shore community composition and functioning. Recent survey data, combined with historical data from 1970, were used to assess temporal changes over the intervening period in rocky shore communities at two sites (Wireless Point and Wireless Island). Three kinds of changes emerged: (1) the appearance of alien species; (2) the effects of increased harvesting pressure; and (3) the direct and indirect effects of these changes on other species. A striking result was transformation of mid-shore zones on exposed shores by the appearance of the invasive Mediterranean mussel *Mytilus galloprovincialis*, and the indirect effects of this on the demography and vertical zonation patterns of the granular limpet *Scutellastra granularis*. Adult limpets have become excluded by the mussel, whereas juveniles find a secondary home on the shells of the mussel and their abundance has increased. To further disentangle the effects of harvesting from those of alien invasions, a spatial comparison was made between two currently unharvested no-take sites (Scarborough South and Scarborough North) and two regularly harvested sites (Kommetjie and Wireless Point). Harvesting has substantially depleted the granite limpet *Cymbula granatina* and Argenville’s limpet *Scutellastra argenvillei*. This has led to the proliferation of opportunistic seaweeds, such as *Ulva* spp. The dual effects of alien invasive species and over-harvesting have major ecosystem effects but do not necessarily diminish biodiversity because the alternative habitats that have developed provide opportunities for colonisation by additional species.

## Introduction

Harvesting of rocky shore organisms has been practiced since the early days of human history but has intensified in recent decades due to increased human population density along coasts, especially in urban areas [1–5]. Increased subsistence, recreational and illegal harvesting of intertidal and shallow-water organisms [2–4, 6] have raised concerns that reducing densities and sizes of harvested species dramatically alters both population and community composition [3–5, 7–9]. To implement appropriate management measures such as Marine Protected Areas (MPAs), authorities need information about the impacts of harvesting on rocky shore fauna and flora [6–8, 10–14] as well as the effects of other drivers of change, such as invasive species [15–19]. In this study, we assess the joint effects of harvesting and invasive species, specifically in the context of South African rocky shores within the Table Mountain National Park, although the insights gained are relevant to management worldwide.

Changes in harvesting pressure have been linked with both direct and indirect effects on rocky shore populations and communities [11–13, 20, 21]. Direct impacts often include declines in the density of harvested species [6, 9, 20, 22–26], but if protection is provided inside MPAs, densities of previously intensely harvested species often increase, as observed for *Fissurella picta*, *Fissurella limbata* [27] and *Concholepas concholepas* [28, 29] in Chile. Harvesting often reduces the sizes of target species specifically, as demonstrated for the limpets *Cymbula oculus* [9], *Lottia gigantea*, and the winkle *Tegula funebralis* [22, 30]; whereas sizes of rarely harvested species would not be expected to differ between protected and harvested areas if harvesting is main factor influencing size. There are exceptions, however. For example, historical comparisons of the sizes of four gastropod molluscs in California has shown that all have declined in size over time, irrespective of whether they are targeted for harvesting or not [31]. The whelk *Nucella lapillus* has been shown to either increase in size in the face of harvesting directed at other species [32], or to decline in size due to the indirect effects of harvesting one of its main food sources, the mussel *Mytilus edulis* [23]. Apart from its effects on individual species, intense harvesting also can change the entire community composition and functioning of rocky shore communities [3, 7, 8, 33].

In addition to harvesting, rocky shores in South Africa and worldwide have been severely affected by invasive alien species, which also alter community composition and the abundances and sizes of indigenous species [15–19, 34, 35]. One of the most notable of these aliens is *Mytilus galloprovincialis*, a globally invasive species that was first recorded in South Africa in 1979 and has since rapidly spread around the coast. It has invaded semi-exposed and wave-exposed areas, where it forms dense mussel beds [15, 16] and out-competes many indigenous species [15, 17, 34–36]. However, invasive species do not have only negative effects on other species. Mussels, for instance, comprise a secondary substratum for epibiotic growth [37] and, by increasing habitat complexity, act as ecosystem engineers that facilitate the survival of other organisms that take advantage of the shelter from waves and predators provided by dense mussel beds [38]. The arrival of *M*. *galloprovincialis* in South Africa has led to an overall increase in densities of the limpet *Scutellastra granularis* in zones dominated by the mussel because it provides a favourable substratum for the limpet’s juveniles [16]; but it has also led to a decrease in the mean size of *S*. *granularis* [18] by excluding adults from the primary rock face. While species-specific effects of interactions with *M*. *galloprovincialis* have been well documented, the influence of *M*. *galloprovincialis* on community composition has received less attention [34, 35].

The aim of this study was to assess the dual effects of harvesting and the invasive alien mussel *Mytilus galloprovincialis* on rocky-shore biodiversity in the Table Mountain National Park Marine Protected Area (hereafter referred to as Table Mountain MPA). Since both pressures have dramatically increased since the 1970s but the MPA provides protection only in terms of harvesting, two comparisons were made: (a) between historical data gathered in 1970 and our own surveys in 2017 at two sites, one of which (Wireless Point) is accessible and intensely harvested and the other (Wireless Island) less accessible but also harvested; (b) among four sites surveyed in 2017, comprising two sites in an inaccessible ‘no-take’ MPA (Scarborough North and South), and two sites that are accessible and experience intense harvesting pressure (Wireless Point and Kommetjie). The paper builds on our previous assessment of the efficacy of no-take zones in the MPA, covering both ecoregions and both of the most common rock types [39]. It has broad relevance to the concepts of habitat, facilitation and trophic cascades which–as both Gribben et al. [40] and Firth et al. [41] point out–have seldom been empirically investigated on rocky shores, despite their relevance to both the effects of alien species and their influence on harvested species.

Five hypotheses were tested: (1) Densities and sizes of the commonly harvested patellid limpets *Cymbula granatina* and *Scutellastra argenvillei* have declined between 1970 and 2017, and greater declines are expected at the easily accessible than the less accessible site due to differences in harvesting levels. (2) The rarely harvested patellid limpet *S*. *granularis* has increased in average density and decreased in average size where *M*. *galloprovincialis* has become dominant, providing an sheltering habitat for juvenile limpets. (3) Community composition has been altered by the dual effects of the invasion of *M*. *galloprovincialis* and increased harvesting in the area over the past decades. (4) In 2017, average densities and sizes of harvested limpets are greater at sites that are fully protected in no-take areas of the MPA than at harvested sites; and (5) Community composition in 2017 differs among sites with different levels of harvesting pressure. By assessing the cumulative effects of harvesting and an invasive species on rocky shores, this study allows assessment of the effectiveness of the Table Mountain no-take sections of the MPA in terms of protecting exploited stocks and coastal biodiversity.

## Materials and methods

### Study area and sites

**Fig 1:**
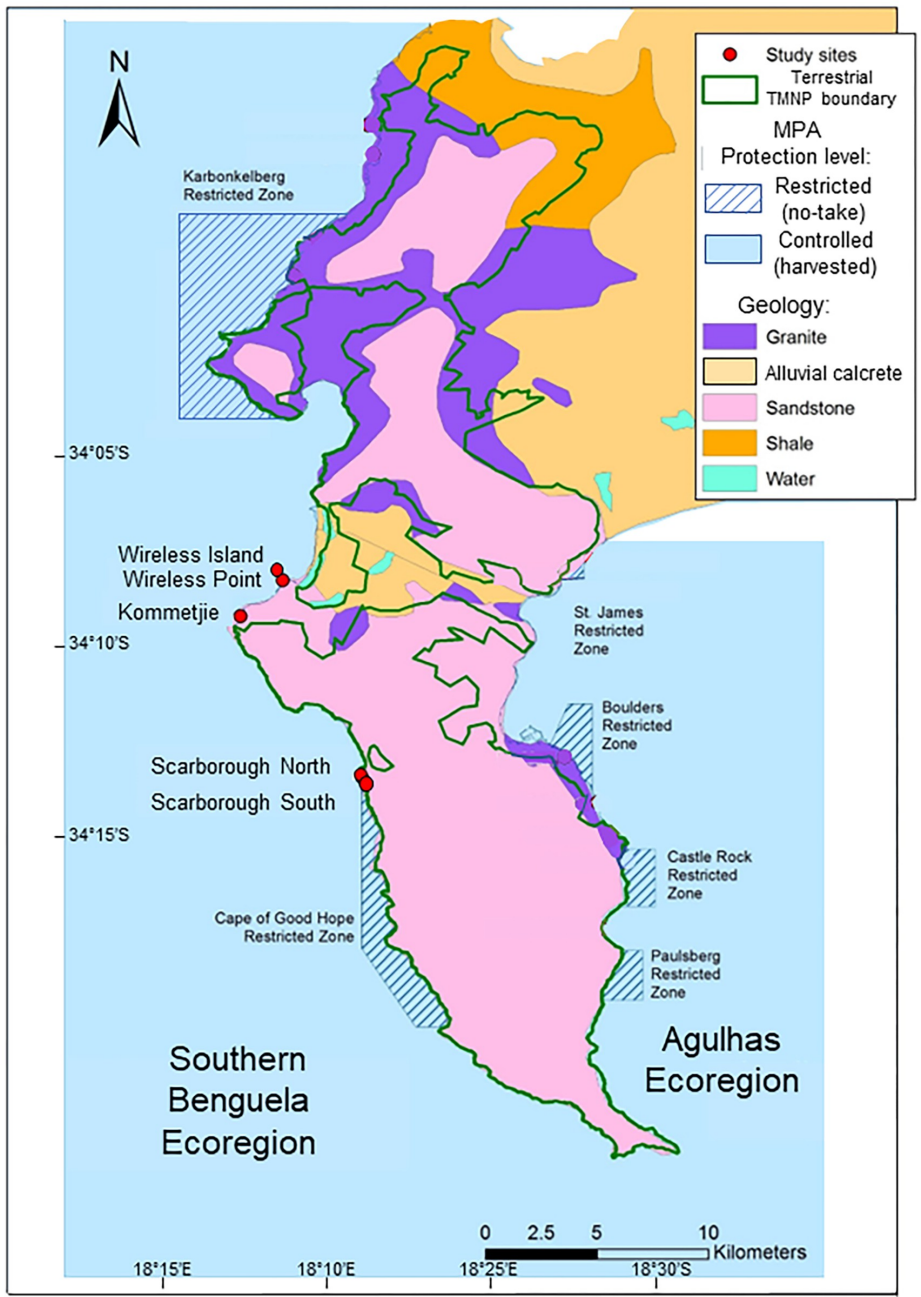
Map of the Table Mountain MPA showing the study sites (red circles). Wireless Island, Wireless Point and Kommetjie are in a ‘controlled’ zone where harvesting is permissible, and Scarborough North and South in a ‘restricted’ no-take zone.

The study was conducted in the Table Mountain National Park MPA (henceforth ‘Table Mountain MPA’) on the coast of the Cape Peninsula, South Africa (Fig 1), which was proclaimed in 2004 and spans two well-defined ecoregions, the warm-temperate Agulhas and the cool-temperate Southern Benguela [42]. The research was conducted under ethical condition prescribed at the time by the University of Cape Town and under permits RES2017-2020/90-DEA issued by the Department of Environmental Affairs, South Africa. The data are archived on the Marine Information Management Systems (MIMS) database for the Department of Forestry Fisheries and the Environment with the following DOI https://doi.org/10.15493/dea.mims.07592023. The MPA has six ‘restricted’ (no-take) areas, which alternate with ‘controlled’ zones where regulated harvesting is permitted, and covers a variety of rock types–most commonly Table Mountain Sandstone and Cape Granite (Fig 1). For this study, comparisons were restricted to sandstone sites of the shores falling in the Southern Benguela Ecoregion, to standardise conditions.

Five study sites were selected: (i) Kommetjie and Wireless Point, which are heavily harvested and fall in controlled-use harvested areas of the MPA, (ii) Wireless Island, also in a controlled-use area, but less frequently harvested because it is less accessible, lying on an emergent reef ca. 25 m offshore from Wireless Point, and (iii) Scarborough North and South, two sites in one of the Cape of Good Hope restricted no-take areas, which are ca. 200 m apart.

The sites were used for different purposes. Historic data were available for only Wireless Island and Wireless Point, so these sites were employed to make temporal comparisons between 1970 and 2017. Wireless Point and Kommetjie (both harvested) and Scarborough North and South (both no-take) were used for spatial comparisons in 2017. In terms of wave action, Wireless Island ranks as ‘exposed’, whereas the other four sites rank as ‘sheltered’ to ‘semi-exposed’ [19]. For this reason, spatial comparisons excluded Wireless Island, and the temporal comparisons were made within each of the Wireless Point and Wireless Island sites. All sites fell within a single upwelling cell [43], thus eliminating upwelling as a variable. For the temporal comparison, there were only two sites for which historical data existed, so replication was limited. For the spatial comparison, we compared two harvested versus two protected sites. Due to the complex geology and biogeography of the region it was, however, not possible to find comparable pairs of sites that were not spatially clustered.

### Sampling for temporal and spatial comparisons

We investigated the dual effects of harvesting and the arrival of the invasive mussel *M*. *galloprovincialis* on rocky-shore biodiversity through temporal and spatial comparisons, following a combination of Before-After and Control-Impact designs [44]. The temporal component compared surveys we did in 2017 with historical data collected in 1970 [GM Branch, unpublished data] at Wireless Island and Wireless Point, before the invasion of *M*. *galloprovincialis* and before an increase in harvesting rates associated with expansion of the coastal human population near this site. Sampling protocols were exactly replicated between 1970 and 2017, and thus considered only the subsets of the community that were recorded in 1970. The spatial comparison focused on surveys in 2017 of two presently harvested and two unharvested sites (see above), which allowed an evaluation of the effects of harvesting on the entire biotic community, using unharvested sites as controls. However, no historical data existed for the no-take sites, which prevented a complete Before-After-Control-Impact (BACI) design.

For the temporal comparisons of community compositions, data were collected by repeating the methods used in 1970, as follows. On sloping platforms, four or five transects parallel to the shore, each with five replicates, were sampled during spring tides, covering four or five shore heights and employing 50 × 50-cm quadrats that were spaced at equidistant intervals up the shore to span the range between the mean low and mean high water marks, in exactly the same locations and shore heights as those surveyed in 1970 (S1A Fig). The quadrats were divided into 25 grid cells, each representing 4% cover, to facilitate accurate estimation of percentage cover. In each quadrat, all species of macro-invertebrates and algae (functionally grouped into corticated algae, encrusting and ephemeral algae) were identified. The percentage covers of sessile organisms were estimated and the numbers of mobile fauna counted. Counts were later converted to percentage cover to standardise the data).

The shell lengths of the commonly harvested *C*. *granatina* and *S*. *argenvillei* and the rarely harvested *S*. *granularis* found in the quadrats were measured using Vernier callipers to an accuracy of 0.1 mm, with a minimum of 50 individuals per zone per species being measured. In the case of *C*. *granatina*, 101 individuals were sampled in 1970, and 50 in 2017.

The historical data provided a means of evaluating changes in density, size and community composition before and after the arrival of alien species and intensification of harvesting.

For the spatial comparisons, separate surveys were conducted at the four sites in four intertidal zones (S1B Fig) characterised by the following dominant species: low shore (*Scutellastra cochlear*); mid shore (*Pachymenia orbitosa*); high shore (*M*. *galloprovincialis* and *Scutellastra granularis*); and top shore (*Scutellastra granularis* and *Porphyra capensis*). Surveys covered the full tidal range (1.8 m), with 15 randomly placed replicate 50 × 50-cm quadrats sampled per zone, but otherwise repeated procedures for the temporal sampling (see above). Again, measurements were made of shell lengths of *C*. *granatina*, *S*. *argenvillei* and *S*. *granularis* (n = 50 per zone in which they were present).

### Data analyses

#### Temporal and spatial comparisons of limpet sizes and densities

To examine the impacts of temporal changes in harvesting on the densities of limpets, the commonly harvested *C*. *granatina* and *S*. *argenvillei* and the rarely harvested *S*. *granularis* were compared between years (1970 vs. 2017) and sites. A two-way ANOVA (with years and sites as fixed factors) was applied for each species. For the same species, to evaluate spatial differences in harvesting among sites in 2017, two-way ANOVAs with fixed factors protection level and site were employed. For temporal comparisons of shell lengths of *C*. *granatina* and *S*. *granularis*, t-tests were applied.

For both temporal and spatial comparisons, the data were first tested for normality and homogeneity of variance using Shapiro-Wilk’s and Levene’s tests, respectively and, where necessary, data were square-root transformed to meet these assumptions. In cases where significant differences were found, Tukey pairwise tests were used as post-hoc tests.

#### Effects of *Mytilus* on zonation of *Scutellastra granularis*

To evaluate the impacts of the invasion of the alien mussel *M*. *galloprovincialis* on the zonation patterns of *S*. *granularis*, two-way ANOVAs (with fixed factors years and zones) were applied to density and size data for this limpet. The data were again tested for normality and homogeneity of variance and square-root transformed to meet the model assumptions. If significant effects were found, Tukey multiple comparison post-hoc tests were used to explore their significance.

#### Temporal changes in community composition

To visualise changes in community composition between years (1970 vs. 2017) and among the five zones, an unconstrained ordination based on Bayesian ordination analysis with two latent variables was fitted to the historical and current community data using the lvsplot in the R package BORAL [45].

To test for statistical differences in percentage cover (taxon-abundance) between years and among zones, we used a full factorial multivariate generalized linear model (manyglm, R package “mvabund” [46, 47]). Adherence to model assumptions was based on plotting the residuals against the fitted model [46]. To probe differences and to identify taxa that were important contributors to differences, univariate tests were additionally run for each taxon, using the *p*.*uni* = “adjusted” argument.

#### Spatial differences in community composition

The Bayesian ordination and generalised linear model-based approaches used in the temporal analysis were not suitable for these analyses since the high number of species (dependent variables) prevented the models from converging. Multivariate percentage cover data for the four sites compared for spatial differences were thus visualised by an ordination using non-metric multidimensional scaling (MDS) performed in PRIMER (Plymouth Routines in Multivariate Ecological Research, Version 6.0).

To statistically investigate differences in community composition among sites of different protection levels, a two-way PERMANOVA with factor ‘site’ nested in ‘protection level’ was performed on the species-abundance data. The nested design was chosen to account for site-specific differences due to local geomorphology. Because the zones reflect different biotopes (i.e. different benthic communities), the analyses were done separately for each zone. Pairwise comparisons were done to test for differences in community composition among sites. Prior to the analysis, data were standardised (by sample) and square-root transformed. To further explore the differences among sites and zones and to establish the species or groups mainly responsible for any observed differences, SIMPER (similarity percentage) analyses were performed. A cumulative cut off of 90% was applied and only taxa contributing >10% to differences are presented.

### Correlations between limpet density and algal cover

To evaluate ecosystem effects of harvesting, we explored relationships among the abundances of all limpets and each algal group (corticated, encrusting and ephemeral algae) by Pearson correlations. Given the dependency of P values on sample size, we included an estimation of the effect size [48], which was considered to be small for correlation coefficient (r) values around 0.1, medium for r ~ 0.3, and large for r ~ 0.5.

## Results

### Effects of harvesting on densities and sizes of limpets

#### Temporal comparisons

**Fig 2:**
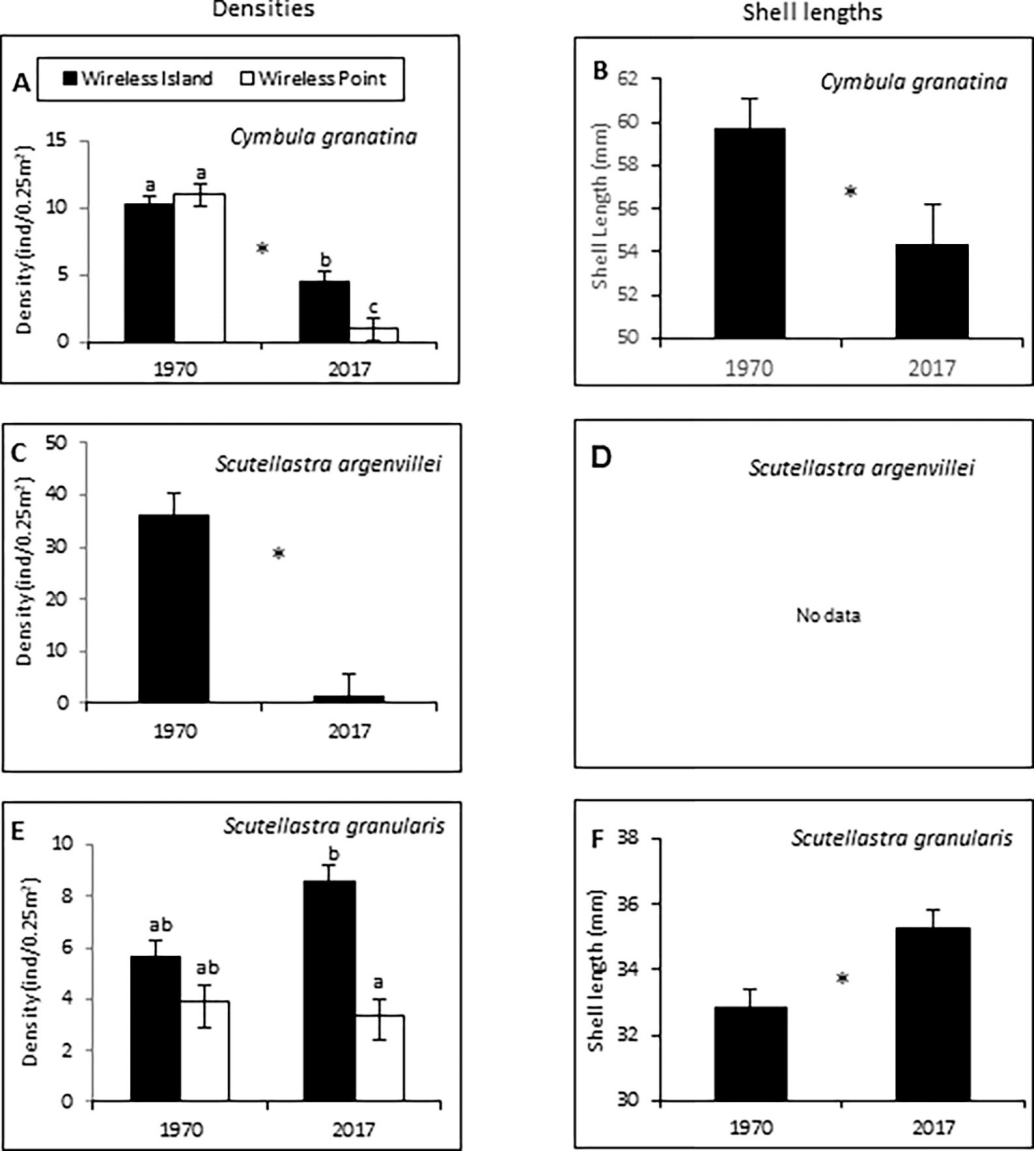
Mean (+1SE) densities (left) and shell lengths (right) in 1970 and 2017, for (A, B) *Cymbula granatina*, (C, D) *Scutellastra argenvillei* and (E, F) *S*. *granularis*. No historical length-data were available for *S*. *argenvillei* and it was absent from Wireless Point. Shell length data for were available for only one site (Wireless Island). Asterisks between years and different letters above error bars indicate significant differences (P < 0.05).

Densities of the frequently harvested limpet *C*. *granatina* were similar at the two sites in 1970, but showed substantial and significant reductions at both sites by 2017. The interaction between year and site was also significant, because the decline at Wireless Point was much greater than at Wireless Island (Fig 2A; S1 Table). The density of the other commonly harvested limpet, *S*. *argenvillei* (present at wave-exposed Wireless Island only), also declined significantly between the years (t = 5.28, df = 9, p < 0.001; Fig 2C). In contrast, densities of the rarely harvested limpet *S*. *granularis* remained constant between years at both sites (Fig 2E; S1 Table).

Shell lengths of *C*. *granatina* and *S*. *granularis* differed significantly between the years at Wireless Island, with the commonly harvested *C*. *granatina* decreasing in size (t = 1.8738, df = 149, p = 0.031), and the rarely harvested *S*. *granularis* increasing (t = -4.769, df = 99, p < 0.001) (Fig 2B and 2F). No historical data existed for sizes of *S*. *argenvillei*, preventing a temporal comparison of sizes for that species (Fig 2D), and for *C*. *granatina* and *S*. *granularis*, historical size data were available only for Wireless Island.

#### Spatial comparisons

**Fig 3:**
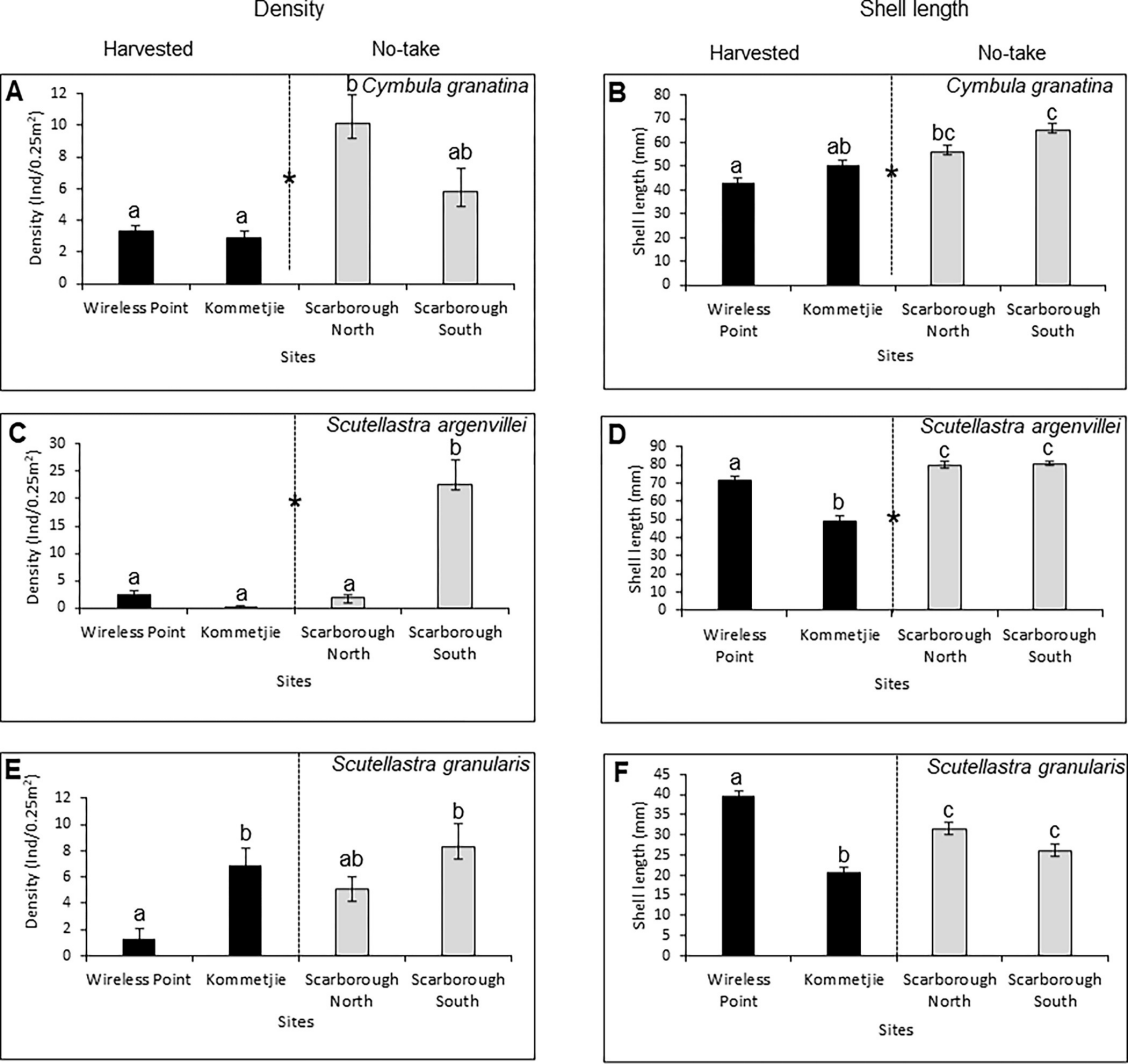
Mean (+1SE) densities (left panels) and shell lengths (right panels) of the limpets (A-B) *Cymbula granatina*, (C-D) *Scutellastra argenvillei* and (E-F) *Scutellastra granularis* at the four study sites. Different letters indicate significant differences according to posthoc tests and refer to comparisons within each panel.

Densities of *C*. *granatina* were significantly less at the harvested areas than in the no-take areas, with the density at Scarborough South being intermediate and not differing significantly from those at the other sites (S2 Table, Fig 3A). Sizes (i.e. shell lengths) of *C*. *granatina* also showed significant effects of protection level and sites (S3 Table; Fig 3B), being smallest at Wireless Point, largest at Scarborough South (the site that lies farthest inside the sanctuary area), and intermediate at Kommetjie and Scarborough North.

*Scutellastra argenvillei* attained greater average densities inside the no-take area, with by far the highest densities found at Scarborough South, while few individuals occurred at the other sites (S2 Table; Fig 3C). The average shell length of this species was unambiguously larger inside the no-take Scarborough sites than in the harvested Wireless Point and Kommetjie sites (S3 Table; Fig 3D).

Densities of the rarely harvested *S*. *granularis* differed significantly among sites, but not between protection levels (S2 Table; Fig 3E). While density was low at Wireless Point, it did not differ statistically from that of Scarborough North, and density at Scarborough South was not statistically different from that at Kommetjie (Fig 3E). Its shell lengths were also not affected by protection levels (S3 Table), with Scarborough North and South being intermediate between the low values at Kommetjie and the high values at Wireless Point (S3 Table; Fig 3F). Mean density thus appeared to be negatively related to mean size, although it was not possible to test this statistically with a sample size of only four sites.

### Effects of *Mytilus* on zonation of *Scutellastra granularis*

**Fig 4:**
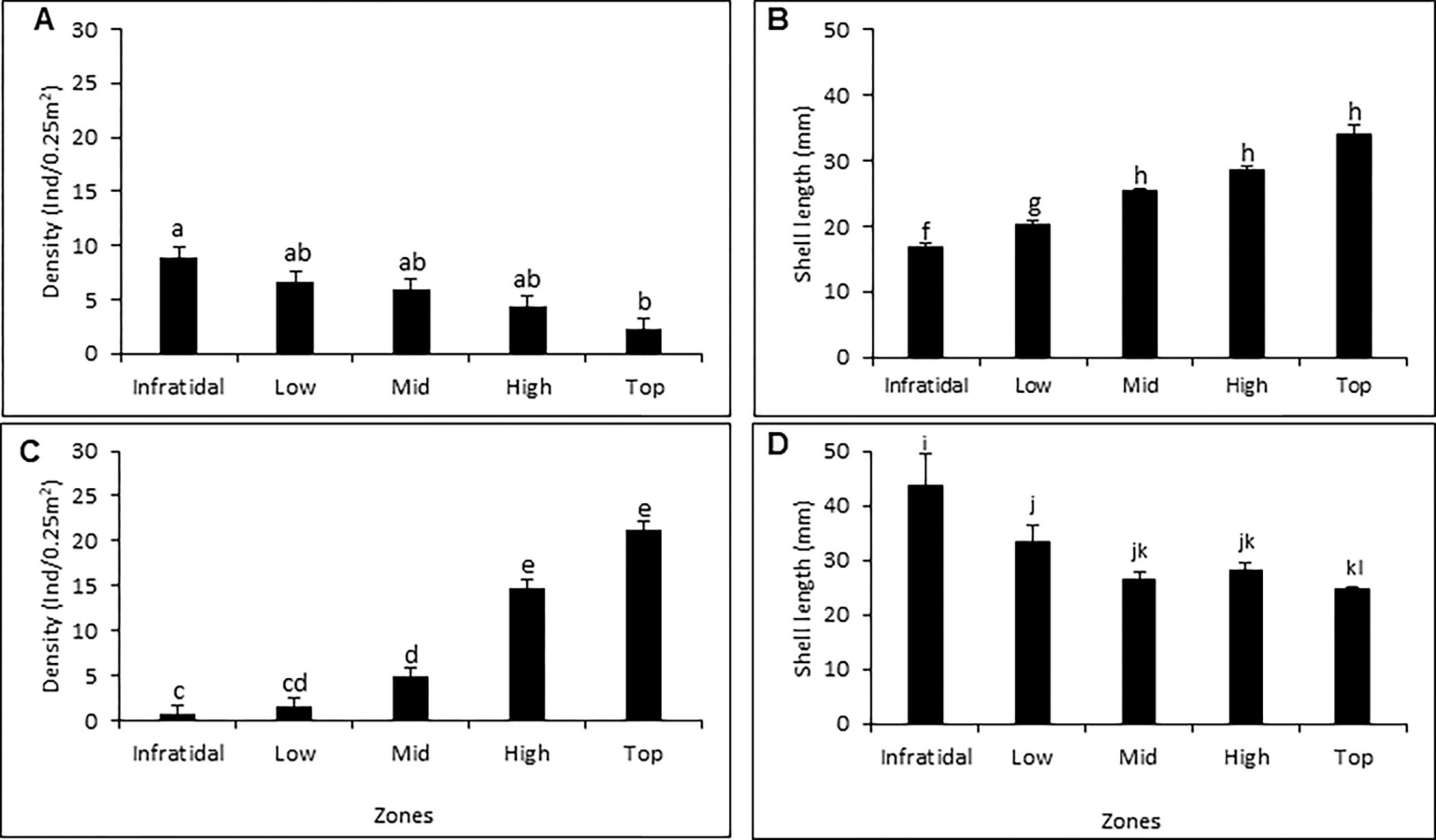
Mean (+1SE) densities (A, C) and sizes (B, D) of *Scutellastra granularis* in different intertidal zones at Wireless Island before the arrival of *Mytilus galloprovincialis* in 1970 (A, B) and after it has invaded in 2017 (C, D). Lower-case letters show Tukey HSD results with different letters indicating significant differences (P < 0.05).

At Wireless Island, *S*. *granularis* exhibited a gradual decrease in densities from the low to the high shore in 1970, whereas in 2017 the opposite trend existed, with densities progressively (and significantly) increasing from the lower to the high shore (Fig 4A and 4C). This led to an absence of any significant effect of time (Years) on mean density, which was explicable by the significant interaction effect of zones and years arising from the reversal of the zonation trends between the years. The reverse pattern emerged for shell length (i.e. sizes) of *S*. *granularis* (S4 Table; Fig 4B and 4D). In 1970, large individuals were recorded higher up on the shore and small individuals lower down, whereas in 2017 the opposite trend was found. Arrival of *M*. *galloprovincialis* (Fig 4A and 4C) thus effectively reversed the previous intertidal zonation patterns for the sizes and densities of this limpet (Fig 4A).

### Changes in community composition

#### Temporal comparisons

**Fig 5:**
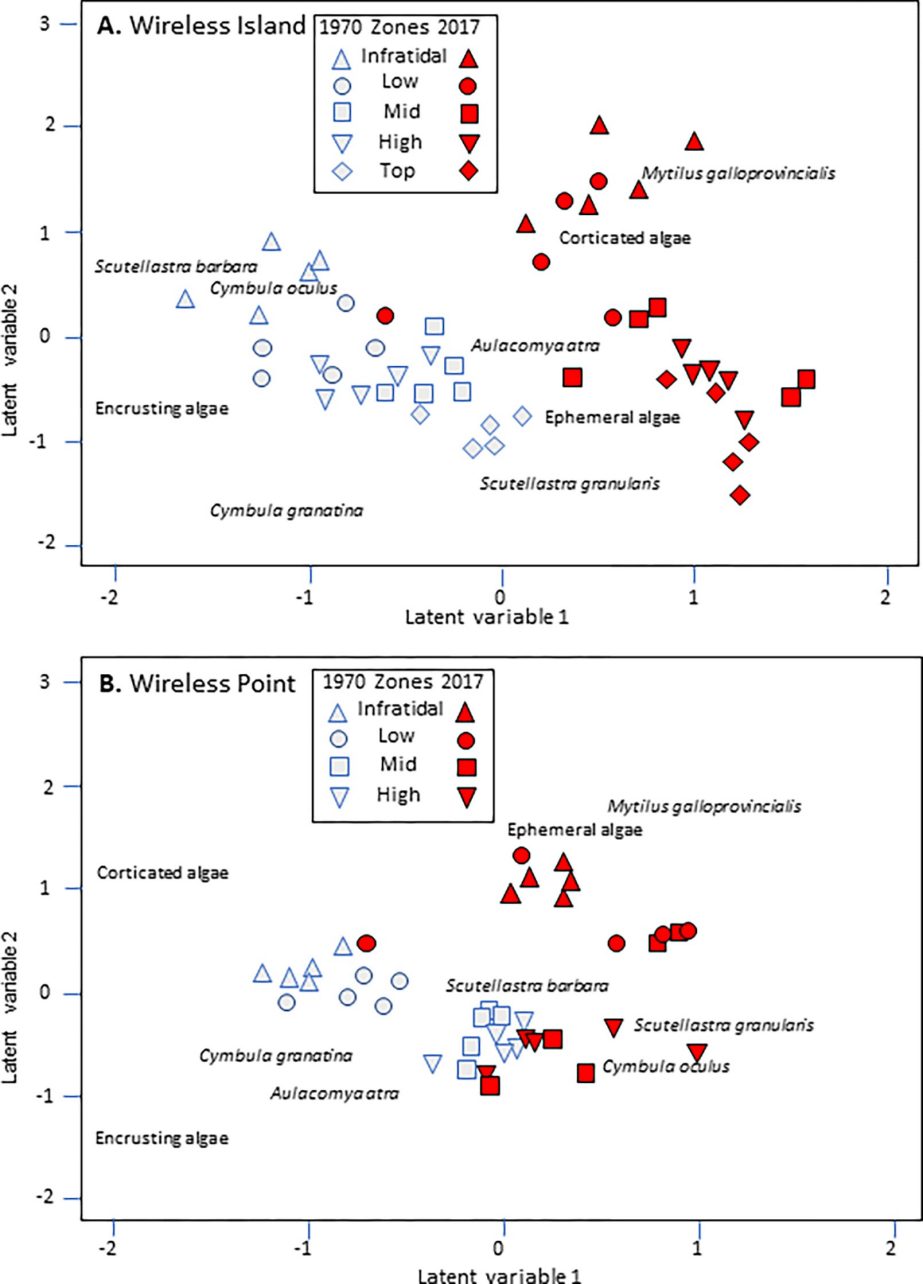
Unconstrained ordination with two latent variables based on taxon abundance in individual quadrats, showing differences between years and different zones (see key), at (A) Wireless Island and (B) Wireless Point. The position of species and functional group names in the ordination portrays indicator species/groups characterising each of the respective years.

Differences in rocky-shore community composition at both sites existed between the years 1970 and 2017 (Fig 5), but the magnitude of the differences varied depending on the intertidal zone considered, as reflected in a significant Year × Zone interaction (S5 Table). At Wireless Island (Fig 5A), two clusters were distinguishable: 1970, characterised by greater abundances of the limpets *S*. *barbara*, *C*. *oculus* and *C*. *granatina* and encrusting algae; and 2017, when corticated and ephemeral algae, the alien mussel *M*. *galloprovincialis* and, to a lesser extent, *S*. *granularis*, were more prevalent. At Wireless Point (Fig 5B) the distinction between years was less clear-cut but still significant, with the 1970 cluster being driven by a greater abundance of encrusting algae, *C*. *granatina* and the ribbed mussel *Aulacomya atra*, and the 2017 cluster by more ephemeral algae, *S*. *granularis* and *C*. *oculus* (Fig 5B).

Exploring changes in the abundances of individual taxa over time (S6 Table) *M*. *galloprovincialis* was originally absent from Wireless Island, but had become well established by 2017, and ephemeral and corticated algae had increased significantly. At the more wave-sheltered and more heavily harvested Wireless Point, *M*. *galloprovincialis* failed to become established. Other effects were confounded by interactions between year and zone, but indicated that *A*. *atra* and *C*. *oculus* declined, whereas ephemeral algae increased. In the case of *A*. *atra* the interaction reflected its disappearance on the low shore and appearance on the high shore; *C*. *oculus* consistently declined but at variable rates among zones; ephemeral algae experienced increases in some but not all zones.

### Spatial comparisons

**Fig 6:**
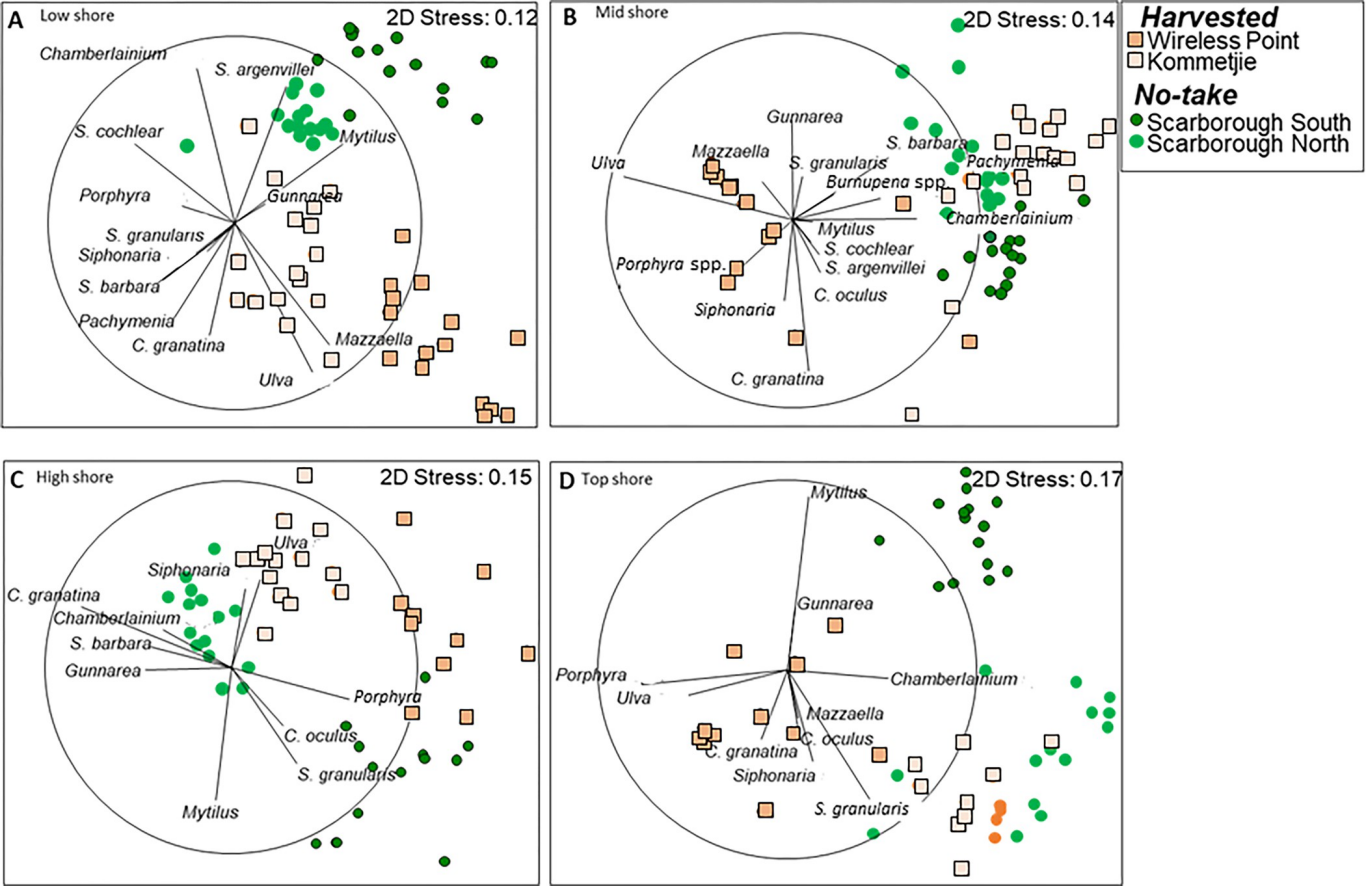
Multidimensional scaling (MDS) of species abundance in the four zones: (A) low shore, (B) mid shore, (C) high shore and (D) top shore, reflecting differences in community composition among harvested (Kommetjie, Wireless Point) and no-take areas (Scarborough North, Scarborough South). Abundances of diagnostic species are presented in multi-dimensional space as well as vectors based on correlations.

Despite significant differences at all shore levels between replicate sites, community composition consistently differed significantly between protection levels (harvested vs. no-take) (S7 Table). Sites of the same harvesting level were generally clustered and separated from those of the opposite level of protection, except in the mid shore where one of the harvested sites (Kommetjie) clustered with the two protected sites (Fig 6A–6D).

**Fig 7:**
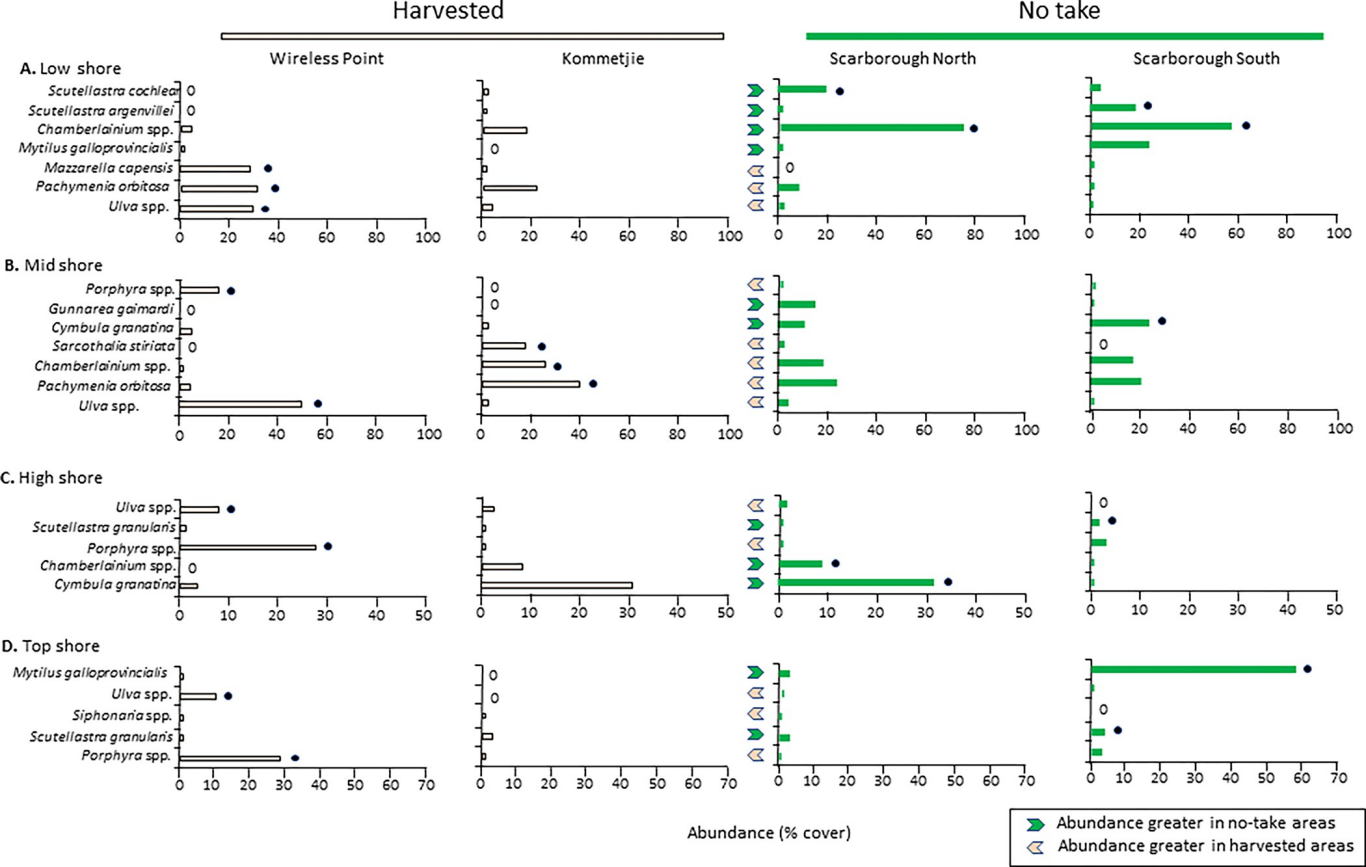
Percentage cover of species responsible for the dissimilarities between the harvested sites (Wireless Point and Kommetjie), and no-take sites (Scarborough South and Scarborough North) in (A) low, (B) mid, (C) high and (D) top zones. Black dots identify species distinguishing sites (based on SIMPER analyses) and are placed in the site with the greater or greatest abundance; 0 = absence. Arrows indicate the direction of abundance of species between harvested and no-take sites.

Species responsible for the major differences in community composition among sites are shown in Fig 6 (black vectors) and Fig 7 (black dots). Macroalgae (*Mazzella capensis*, *Pachymenia orbitosa*, *Ulva* spp. and sometimes *Porphyra* spp.) prevailed at the harvested sites of Wireless Point and Kommetjie. In contrast, the two no-take Scarborough sites displayed greater cover of *Mytilus galloprovincialis*, especially in the top and high shores, and most obviously at Scarborough South. Dense beds of *C*. *granatina* in the midshore discriminated the protected Scarborough sites from those of the harvested sites at Wireless Point and Kommetjie, and were also prevalent in the high shore at Scarborough North. Two limpets, *S*. *cochlear* and *S*. *argenvillei*, together with their associated encrusting algae *Chamberlainium* spp., prevailed in the low shore at the protected Scarborough sites while being less common at the harvested Wireless Point and Kommetjie sites. *Scutellastra granularis* was present at equal abundance at protected and harvested shores in the high and top zones at most shores. Notable was the fact that *Siphonaria* spp. grouped with species associated with the two harvested shores at all four shore levels (Fig 6).

### Correlations between limpet density and algal cover

A number of trends emerged from correlations between limpet densities and percentage cover of different algal groups, despite a fair amount of variance in the data. Effect sizes were medium for ephemeral (r = -0.30) and corticated (r = +0.39) algae and large for encrusting algae (r = +0.54) (S2 Fig). We were thus confident that these relationships are real and not just a result of large sample sizes. Cover of ephemeral algae declined with an increase in limpet abundance, being highest in areas with <3% cover of limpets. Corticated algae displayed an unexpected parabolic relationship with limpet density, declining at both low and high densities of limpets. Encrusting algae displayed a linear positive relationship with limpet densities. All these patterns must be treated with caution because of the magnitude of the variance and because of violations of homogeneity of residuals, so we used these results to interpret trends in the data and not for exact predictions.

## Discussion

Our central aim was to assess the impacts of harvesting and the arrival of an invasive mussel on (a) limpet sizes and abundances and (b) rocky-shore community composition in the Table Mountain MPA, through a combination of temporal and spatial comparisons.

The temporal comparison between 1970 and 2017 at two sites where harvesting pressure has multiplied and the invasive mussel *M*. *galloprovincialis* has invaded since the 1970s revealed major changes in both the densities and sizes of harvested limpets and community composition. Densities of the two most-commonly harvested limpets, *Cymbula granatina* and *Scutellastra argenvillei*, declined markedly. A secondary effect of their depletion was a proliferation of macroalgae. In contrast, densities of the rarely harvested *Scutellastra granularis* remained unchanged. Another striking finding was a reversal of the zonation pattern previously exhibited by *S*. *granularis*, clearly associated with the establishment of the alien mussel *M*. *galloprovincialis*.

Spatial comparisons made in 2017 between the two protected sites and the two harvested sites revealed densities and sizes of *C*. *granatina* and *S*. *argenvillei* were greater at the no-take sites, while variability for the rarely harvested *S*. *granularis* was unrelated to harvesting levels, reinforcing the interpretation that harvesting drove reduction in harvested limpets. Marked differences in community composition existed between harvesting levels (despite significant among-site variability). Macroalgae were consistently more abundant at harvested than protected sites, which is a typical community response to harvesting of grazers.

Differences among sites are not unexpected, given that significant differences in community composition have frequently been noted among sites on rocky shores, even when they are separated by short distance of 10s of km [49–51], and have been attributed to variations in recruitment [52], linked to differences in factors such as wave action and upwelling [53, 54]. However, in our study, all the sites we examined fell within a single upwelling cell [53] and we standardised wave action by selecting shores within a limited range of wave action conditions. The contrasts we recorded in space and in time are thus more probably linked to differences and changes in harvesting intensity, and the arrival and spread of the alien mussel *M*. *galloprovincialis*.

### Effects of harvesting

#### Temporal comparisons of densities of limpets

The densities of commonly harvested limpet species declined substantially over the years. Previously, populations of *C*. *granatina* and *S*. *argenvillei* formed dense beds with densities of respectively 70-288/m^2^ and 162-216/m^2^, which equate well with those at other unharvested sites [17, 19, 55, 56]. Currently, the populations of *C*. *granatina* and *S*. *argenvillei* at Wireless Point and Wireless Island have declined by 56% and 97% respectively. These declines far exceed those reported elsewhere for experimental thinning of *C*. *granatina* and *S*. *argenvillei*, which were reduced respectively down to 33.4% and 25% of their original densities for individuals ≥60 mm and ≥75mm [17]: yet even at those less severe levels of depletion, ecosystem effects were detectable [55]. The depletion levels we recorded are more comparable to those reported for other limpet species considered to be heavily harvested, such as 50% for *S*. *longicosta* [57] and 75% for *C*. *oculus* [9] on the east coast of South Africa, decimation and the threat of global extinction of *Scutellastra mexicana* in the eastern Pacific [58–60] and the >90% reduction and local extinctions of *Patella ferruginea* in the Mediterranean [59] and *Patella candei* in the Canary Islands [61]. In contrast, the rarely harvested *S*. *granularis* displayed a (non-significant) average 25% increase in density over the years, supporting the idea that harvesting was the main cause of the declines of harvested species. Intensification of harvesting is attributable to a rise in human populations in the vicinity of Cape Town [2, 43] and near the Wireless study sites in particular, where the steadily growing low-income settlement of Masiphumelele was established in the 1980s.

Comparing the two harvested sites, the significantly greater densities of *C*. *granatina* at Wireless Island, compared to Wireless Point, probably reflects relatively low harvesting pressure at Wireless Island because it is detached from the mainland and less accessible. Inaccessibility of rocky shores is well known as a factor that reduces harvesting pressure on intertidal gastropods [4, 62–65]. In line with this, greater densities of *Phorcus turbinatus*, *Patella ferruginea* and *Patella ulyssiponensis* have been reported on inaccessible islands compared to accessible mainland sites in Italy [26, 66, 67], and for *Haliotis spadicea* and *Scutellastra longicosta* in South Africa [68, 69], the mussel *M*. *galloprovincialis* in Portugal [70, 71] and the alga *Durvillaea antarctica* in Chile [72]. However, the higher density of *S*. *granularis* at Wireless Island than at Wireless Point may be because it is indirectly benefitting from the greater exposure to wave action there, as the alien mussel *M*. *galloprovincialis* reaches greatest densities in areas of strong wave action [73], and provides secondary habitat that boosts the numbers of juveniles of *S*. *granularis* [15, 16, 18, 34, 35].

#### Temporal comparisons of shell lengths of limpets

The decline in shell lengths of the commonly harvested *C*. *granatina* over time is consistent with effects of harvesting because large individuals are targeted as food [1, 22, 31, 74], a phenonemon that has been recorded even from prehistoric times [75, 76]. Declines in shell lengths of harvested species, e.g. *Lottia gigantea* [31], *Patella caerulea* (as *P*. *candei crenata*), *Patella aspera* [74] and *Tegula funebralis* [22], are common worldwide, especially in areas near residential developments that are frequently visited by shellfish gatherers [74]. However, other factors such as competition and resultant declines of food sources can also reduce average sizes of limpets [77]. Even gastropods that are not harvested, such as *Nucella lapillus* and *Siphonaria lessonii*, may decrease in size if food availability is reduced by competition [23, 78]. However, competition is unlikely to explain the reduction in size of *C*. *granatina* since its size reduced in parallel with a reduction in density. In its adult stage it becomes a ‘trapper’ that relies on trapping drift algae and is therefore relatively independent of local epilithic algal supplies [56]. Moreover, *S*. *granularis*, which is rarely harvested, increased in shell length over time in the same area. When harvesting was prevented in an MPA in Chile, the shell lengths of the two commonly harvested limpets *Fissurella picta* and *L*. *gigantea* increased despite their density rising [78]. These observations strongly indicate that harvesting, not competition, caused the declines in sizes of harvested species. These facts, together with the observation that sizes of *S*. *granularis* increased over time–likely as a result of reduced competition from harvested limpets–strengthen the argument that harvesting was the cause of decline in shell lengths of *C*. *granatina*.

#### Spatial differences in densities of limpets

Protection played a major role in determining the densities of the commonly harvested *C*. *granatina* and *S*. *argenvillei*. The higher densities of *C*. *granatina* in the Scarborough no-take areas compared to the harvested Wireless Point and Kommetjie sites indicates it is benefiting from the greater protection in no-take areas, as has been demonstrated for limpet populations elsewhere [8, 9, 22, 79]. The case for *S*. *argenvillei* is less clear-cut, as its abundances were greater at only one of the protected sites. The four- to 15-fold differences in densities of *C*. *granatina* indicate that the no-take areas in the reserve succeed in maintaining the exceptionally high natural densities that this species achieves in other unharvested parts of the west coast [56]; and this is probably true for *S*. *argenvillei* as well. Similarly, higher densities have been reported for *Cymbula oculus* [9, 57], *Perna perna* [80] and *S*. *longicosta* [57] in MPAs on the South-east coast of South Africa, and for *Patella ferruginea* in Sinis-Isola MPA, Italy [67], following exclusion of harvesting from rocky shores.

The lack of protection effects for *S*. *granularis* was expected as this species was not collected by harvesters [39]. The low density of *S*. *granularis* at Wireless Point compared to Kommetjie and Scarborough South was, however, unexpected. Density of this species is enhanced in areas with a high cover of *M*. *galloprovincialis*, due to improved recruitment and survival of juveniles of the limpet in mussel beds [15, 16, 18, 34, 35]. *Mytilus galloprovincialis* was almost absent from Wireless Point. Differences in the densities of *S*. *granularis* may therefore be a secondary effect of the relative abundance of *M*. *galloprovincialis*, and unrelated to differences in harvesting. The most likely explanation for the scarcity of *M*. *galloprovincialis* at Wireless Point is that this site lies in the lee of Wireless Island and therefore experiences less wave action, and wave action supplies particulate food for the mussel [73]. However, the reasons for the high abundance of *S*. *granularis* at Kommetjie, where *M*. *galloprovincialis* was also scarce, remain unclear.

#### Spatial differences in shell lengths of limpets

Because of the common practice for harvesters to select large individuals, mean sizes of targeted species are frequently reduced on harvested shores because of the truncation of larger sizes [9, 10, 30, 31, 33, 69, 81]. The presence of larger *C*. *granatina* and *S*. *argenvillei* at the protected Scarborough sites than at the harvested sites of Kommetjie and Wireless Point accords with size-selective harvesting [39]. The argument that harvesting is the root cause of this pattern in these species is supported by the fact that the rarely harvested *S*. *granularis* did not experience a reduction of size in harvested areas: indeed, its sizes at one of the harvested sites, Wireless Point, were 12–19% greater than at either of the protected sites. The differences in shell lengths of *C*. *granatina* and *S*. *argenvillei* translate to 8–12% declines, falling within the range of declines reported for other molluscan populations under intense harvesting pressure: e.g. 8.6–10% for *Anadara scapha*, *Gafrarium tumidum* and *Modiolus auriculatus* in New Caledonia [10, 11], 20–30% for *C*. *oculus* in South Africa [9] and 28% for *Lottia gigantea* in California [30].

### Effects of *Mytilus*

#### Temporal changes in the zonation of *Scutellastra granularis*

Our study is the first to report the effect of the alien *M*. *galloprovincialis* on the zonation patterns of *S*. *granularis*. Invasion by this mussel has shifted the centre of recruitment of *S*. *granularis* from the infratidal and bottom zones to the mid and high shore. Before the arrival of *M*. *galloprovincialis*, densities of *S*. *granularis* decreased upshore and its average size increased upshore–to the extent that Branch [77] classed it as one of a group of ‘migratory’ species that settle low on the shore and then shift progressively upshore–presumably escaping intense low-shore competition as it ages and growth diminishes the threat of desiccation [82]. The arrival of *M*. *galloprovincialis* increased densities of this limpet at Wireless Island, but concentrated its recruits in the mussel beds, with a downward movement of adults as they shifted from the mussel beds to vacant space below the beds. These findings agree with results of others who have reported lower densities of *S*. *granularis* where *M*. *galloprovincialis* is absent or scarce along the west coast, and reductions in the abundance of *S*. *granularis* in the infratidal zone after arrival of *M*. *galloprovincialis* [15, 16, 18, 34, 35]. Facilitation of gastropods by mussels is not unique to the sites where we worked. For example, in Chile the limpet *Siphonaria lessoni* and the whelk *Nacella magellanica* attain higher densities in beds of the mussel *Perumytilus purpuratus* than on bare rock [83].

Prior to the arrival of *M*. *galloprovincialis*, the zonation of *S*. *granularis* reflected a pattern typical of many ‘upshore’ species, such as *Lottia digitalis*, *Collisella subrugosa*, *Helcion concolor* or *Siphonaria guamensis*, for which recruitment occurs low on the shore and individuals migrate upshore as they become larger [1, 77, 84–86]. However, the invasion by *M*. *galloprovincialis* has altered the size zonation patterns of *S*. *granularis*: recruits and juveniles are now found predominantly in the mid to upper zones and adults in the infratidal zone. *Mytilus galloprovincialis* has ameliorated the previously harsh conditions on the high shore, which usually limit settlement (or survival) of recruits to the low shore, so that *M*. *galloprovincialis* promotes survival of recruits and juveniles of *S*. *granularis* [16, 34, 35]. Similar interactions have been observed on the south coast of South Africa, between *S*. *granularis* juveniles and barnacles, where high-shore barnacles moderate physical conditions, increasing recruitment of *S*. *granularis* there, but decreasing its size [87]. The presence of large-sized *S*. *granularis* in the infratidal zone indicates a downward migration of individuals as they grow into the zone where there are few individuals of *M*. *galloprovincialis*. Previous studies have shown that adult *S*. *granularis* inhabit patches of bare rock that are devoid of *M*. *galloprovincialis* and small ones predominate in mussel beds [18]. The downwards movement may reduce competition for space in the high shore where primary space suitable for adult *S*. *granularis* is limited, and algal food supplies reduced. Experimental removal of *M*. *galloprovincialis* results in an increase in the density of adult *S*. *granularis* on the primary space that becomes available [16]. Tanaka et al. [86] reported a similar positive correlation between the amount of bare rock and the sizes of the limpet *Collisella subrugosa* on the high shores of Brazil. This demonstrates the need for adequate primary space by adult limpets. Limitations on space availability on the mid- to upper zones due to monopolisation by *M*. *galloprovincialis* have forced adult limpets to reverse their previous upshore migratory behaviour [77].

### Community responses

#### Temporal changes in community composition

Substantial changes in community composition took place between 1970 and 2017, for which there are several explanations. One obvious agent of change is the arrival and establishment of the alien Mediterranean mussel *M*. *galloprovincialis*, which drives shifts in rocky-shore community composition in South Africa in a number of ways [34, 35]. Due to its high tolerance of desiccation, high growth rate and fecundity, *M*. *galloprovincialis* monopolises mid to high zones and creates a complex habitat that reduces physical stress and supports a rich interstitial fauna [18, 19, 34, 35, 88]. The secondary habitat formed by this mussel increases the density of *S*. *granularis* in the top and high zones and while reducing its densities in the infratidal and low zones where they were previously most abundant. Following the arrival of *M*. *galloprovincialis*, increases in the abundance of *S*. *granularis* juveniles in mussel beds and diminishment of adults on the shrinking primary space have previously been reported on the west coast of South Africa [15, 16, 18, 34, 35].

A second obvious contributor to the observed changes in community composition over time is the intensification of harvesting, which was non-existent or minimal at the surveyed sites in 1970 and has since become a common practice at the two Wireless sites (and at Kommetjie) [39]. A reduction in targeted limpet species and increases in various macroalgal groups that proliferate under reduced grazing pressure are common effects of harvesting [7, 21, 33, 89–96]. The types of algae that came to predominate differed among sites, with ephemeral species increasing at Wireless Point and corticated species at Wireless Island. This may reflect differences in the abundances of limpets, thus influencing the types and amounts of algae that develop [20, 92, 94, 97–102]. *Cymbula granatina* used to be common at both sites but was depleted to a greater extent at the more easily accessible Wireless Point, where only few individuals remain on the low shore, and ephemeral algae have taken over the intertidal zone. The drastic decline in numbers of *C*. *granatina* is unlikely due to the arrival of *M*. *galloprovincialis*, as this mussel largely inhabits mid to high zones and prevails on exposed shores [19], whereas this limpet mainly occupies the low shore on sheltered to semi-exposed shores where it can trap drift algae [56]. Thus, the decline in *C*. *granatina* can more likely be ascribed to increases in the human population adjacent to the rocky shore and associated intensification of harvesting [2, 39].

Reductions in the densities of limpets affect not only algae, but predators that eat limpets. It has even been suggested that a contributory factor to the extinction of the Canarian Black Oystercatcher *Haematopus meadewaldoi* was over-exploitation of limpets [103]. Conversely, arrival of *M*. *galloprovincialis* in South Africa has boosted food supplies for the African Black Oystercatcher *H*. *moquini* and contributed to its rebounding population [104, 105].

#### Spatial differences in community composition

Differences in the community composition between rocky-shore sites experiencing high harvesting pressure and those protected from harvesting have been reported many times in South Africa and worldwide [7, 11, 33, 95, 106–108]. Experimental removal of limpets has also often demonstrated this effect [96, 100], leading to increases in macroalgae. Increased macroalgal cover may in turn inhibit the settlement and recruitment of species that require primary substrate or a cover of crustose algae to settle, while increasing settlement of those species that live within algal beds. This has been observed in MPAs along the south and east coasts of South Africa, where protection elevates the abundance of sessile fauna e.g. barnacles and mussels, whereas algal mats are more abundant at harvested sites [7]. Similar to these studies, the harvested sites we surveyed (Wireless Point and Kommetjie) were dominated by extensive mats of *Ulva* spp., *Pachymenia orbitosa* and *Mazzaella capensis*, linked to the to the depletion of limpets such as *C*. *granatina* (and, at Wireless Island, *S*. *argenvillei*), creating opportunities for growth of opportunistic algae like *Ulva* spp. [7, 33, 106, 107]. The dominance of a single genus (*Ulva*) in the community of Wireless Point while at the other harvested site, Kommetjie, *P*. *orbitosa* and *Sarcothalia stiriata* were abundant suggests different degrees of disturbance between these sites. Dominance by opportunistic algae such as *Ulva* spp. reflects a shift between herbivores and algae, with shellfish-gathering increasing algal mats, while protection against harvesting strengthens the limpet/algal interaction, as reflected in the rarity of opportunistic algae at the Scarborough sites. Increased algal growth may have knock-on consequences for the functioning of the ecosystem, including reduced feeding and altered settlement of mussels, and smothering of other organisms through overgrowth [7, 33, 106, 107].

The differences in community composition between sites that experience high versus low harvesting pressure are usually due to commonly harvested species being abundant in protected sites, whereas at harvested sites algae and algal-associated species become distinguishing taxa [7, 8, 33, 106]. For example, Deepananda and Macusi [97] reported dominance of opportunistic algae such as *Gracilaria cassa*, *Valoniopsis pachynema* and *Padina boergesenii* in the community composition of harvested sites in Sri Lanka. Hence, the abundance of *Ulva* spp., an opportunistic species, at the harvested sites we examined was not surprising, and is a common response in areas where grazers have been depleted [20, 90, 92, 94, 98–100].

The greater abundances of *C*. *granatina* and *S*. *argenvillei* at the protected sites we surveyed (Scarborough North and South) indicates direct benefits of protection through no-take areas in this MPA. Similarly, the greater contributions of *M*. *galloprovincialis* to community composition at the protected Scarborough sites showed that this alien species is (ironically) also benefitting from the protection from harvesting. The greater contributions of the crustose coralline alga *Chamberlainium* spp. at the two protected sites reflect a strong association between *Chamberlainium* spp. and *Scutellastra cochlear* [109, 110], as average densities of this limpet were also greater in the protected sites, even although it is not harvested. It is also a reflection of a more general positive association between encrusting coralline algae and limpets, which has been well established in North America [111, 112] as well as on the west coast of South Africa [110, 113].

The predominant occurrence of *Siphonaria* spp. at harvested sites likely reflects a reduction in competition from patellid limpets, as several experimental tests have shown that siphonariids are inferior competitors to patellid limpets [114] due to their less efficient radulae and removal of food [115, 116].

Another prominent driver of decadal changes in community composition on rocky shores is climate change. Our analysis does not probe this, but geographic shifts of distribution and changes in abundance on the coast of South Africa have been securely linked to climate change for several species, including the kelp *Ecklonia maxima* [117], the rock lobster *Jasus lalandii* [118], and the brown mussel *Perna perna* [119]. These shifts have been attributed to changes in the intensity of south-easterly winds, leading to a cooling of nearshore waters on the south-east coast, and to increases in low-oxygen events on the west coast [43, 119].

Comparable dramatic cascading temporal and spatial effects have been demonstrated in Tasmania, where an expansion of the range of the urchin *Centrostephanus rodgersii* has been attributed to a combination climate change and overfishing of predatory fish [120, 121]. With the appearance of this urchin in Tasmania, and the subsequent increase in its abundance, kelp beds have been transformed into urchin barrens, with consequent diminishment of energy flow and biodiversity. Such urchin effects are widespread, as is apparent from a global analysis of urchin dynamics [122]. Our data similarly reveal considerable temporal and spatial changes in community composition and functioning that are related, in this case, to both harvesting of a key grazer and the effects of a dominant invasive alien, *Mytilus galloprovincialis*, with cascading effects up and down the food web, associated with alteration of habitat structure and ensuing effects, as we outline below.

#### Correlations between limpet densities and algal cover

The negative correlation that emerged between limpets and ephemeral algae is not new: many previous studies have shown that removal of limpets increases ephemeral algae [94, 98, 99]. In our study, the highest abundances of these algae were at Wireless Point, where there were relatively few limpets compared to Kommetjie and Scarborough.

The parabolic relationship between corticated algae and limpet densities is more difficult to explain, although it is possible to speculate that disturbance (grazing) is a cause of low abundance at high limpet densities, and competition with opportunistic algae displaces them at low limpet densities.

The positive correlation between encrusting algae (dominated by *Chamberlainium* spp.) and limpets, particularly *Scutellastra cochlear* [123] is partially fuelled by the release of nutrients by *S*. *cochlear*, which are taken up by the surrounding algal ‘gardens’ this limpet occupies [124]. Encrusting algae constitute 80% of the diet of this limpet, and grazing intensity on the thalli promotes their growth rate [110, 124]. In addition, limpet grazing promotes encrusting corallines by preventing competitive overgrowth by foliar algae [110]. Steneck [112] has argued for a mutual dependency between the encrusting alga *Clathromorphum circumscriptum* and the limpet *Acmaea testudinalis*. Maneveldt and Keats [109] noted an absence of encrusting algae in the lower zones of west-coast areas where ever *S*. *cochlear* is absent. A similar positive relationship exists between encrusting algae and *Patella aspera* in Italy [125]. More recently, a mutualistic relationship between the limpet *Patella ulyssiponensis* and crustose corallines has been established in northwest Europe [41], raising the likelihood that the phenomenon is more widespread than is generally appreciated. This strengthens the argument that encrusting algae benefit from coexistence with grazers, particularly *S*. *cochlear* in this case.

Encrusting coralline algae have been shown to be preferential settlement sites for a range of invertebrates, including abalone *Haliotis* spp. [126] and the commercially important *H*. *midae* [127, 128]. Reductions in the abundance of grazers, with resultant effects on encrusting corallines, are therefore of more than academic interest.

## Conclusions

The community composition on the rocky shores of Wireless Island and Wireless Point has changed considerably between 1970 and 2017, driven by arrival of the alien mussel *M*. *galloprovincialis* and intensification of harvesting. Harvesting has decimated the populations of *C*. *granatina* and, to a lesser extent, *S*. *argenvillei*, and reduced the average sizes of both species. In turn, this has increased ephemeral macroalgae, particularly at Wireless Point where they have overrun the shore.

Arrival of *M*. *galloprovincialis* has also changed the abundance of *S*. *granularis* in different zones, reversing the previous density- and size-related zonation patterns. In our spatial comparison of sites, the community composition differed between protection levels and among sites, with communities inside no-take areas being dominated by the commonly harvested taxa *C*. *granatina*, *M*. *galloprovincialis* and *S*. *argenvillei*, whereas at the two harvested sites communities were characterised by greater abundances of algae and the rarely harvested limpet *S*. *granularis*, reflecting the direct and indirect impacts of harvesting versus protection. Whereas harvesting led to lower population densities and smaller sizes of commonly harvested limpets, the rarely harvested *S*. *granularis* did not differ in any systematic way in density or size between the harvested and protected sites, supporting the view that harvesting is the driving factor of the observed differences between the restricted and controlled zones of the Table Mountain MPA.

## Supporting information

S1 Fig Sampling design for (A) temporal comparison at Wireless Island and Wireless Point (Before-After component, 1970 vs. 2017) and (B) spatial comparisons of four sites (Control-Impact component of sites in 2017). MHWS = mean high water springs and MLWS = mean low water springs. Squares indicate quadrats.
(TIF)

S2 Fig Relationships between the density of limpets and (A) ephemeral algae, (B) corticated algae and (C) encrusting algae and supporting statistics from regression analyses (for x+1 values in case of (A)).
(TIF)

S1 Table Two-way ANOVAs of the densities of *C*. *granatina* and *S*. *granularis* comparing temporal differences with factors site and time, and their interaction. Asterisks indicate significant effects.
(DOCX)

S2 Table Results of two-way nested ANOVAs of the densities of *C*. *granatina*, *S*. *argenvillei* and *S*. *granularis* with factors protection level and site (nested in protection level). Asterisks indicate significant effects.
(DOCX)

S3 Table Results of two-way nested ANOVAs of the sizes (shell lengths) of *C*. *granatina*, *S*. *argenvillei* and *S*. *granularis* with factors protection level and site nested in protection level. Asterisks indicate significant effects.
(DOCX)

S4 Table Two-way ANOVA of the densities and sizes of *Scutellastra granularis* with factors year and zones, and their interaction. Asterisks indicate significant effects.
(DOCX)

S5 Table Multivariate generalized linear model analysis of the community composition to test for differences between years and among intertidal zones, and their interaction. Asterisks indicate significant effects.
(DOCX)

S6 Table Univariate tests for differences in abundance over time and among intertidal zones. P-values are adjusted for multiple testing using a step-down resampling algorithm (Wang et al. 2012). Goodness-of-fit of the models is indicated by the residual deviance (Dev), with lower values indicating better fit. Changes in the abundance over time are shown as increases (+), decreases (-) or no change (0). Blank cells = absence or scarcity that prevented analysis.
(DOCX)

S7 Table Two-way nested PERMANOVA with factors protection level (harvested vs. no-take) and site (nested in protection level) showing their effects on community composition in the four intertidal zones. (DOCX)
