# Supplementary figures and images for: Effects of harvesting and an invasive mussel on intertidal rocky shore communities based on historical and spatial comparisons
Source: PLoS One. 2024 Feb 8;19(2):e0294404. doi: 10.1371/journal.pone.0294404 (PMC10852263; doi:10.1371/journal.pone.0294404)

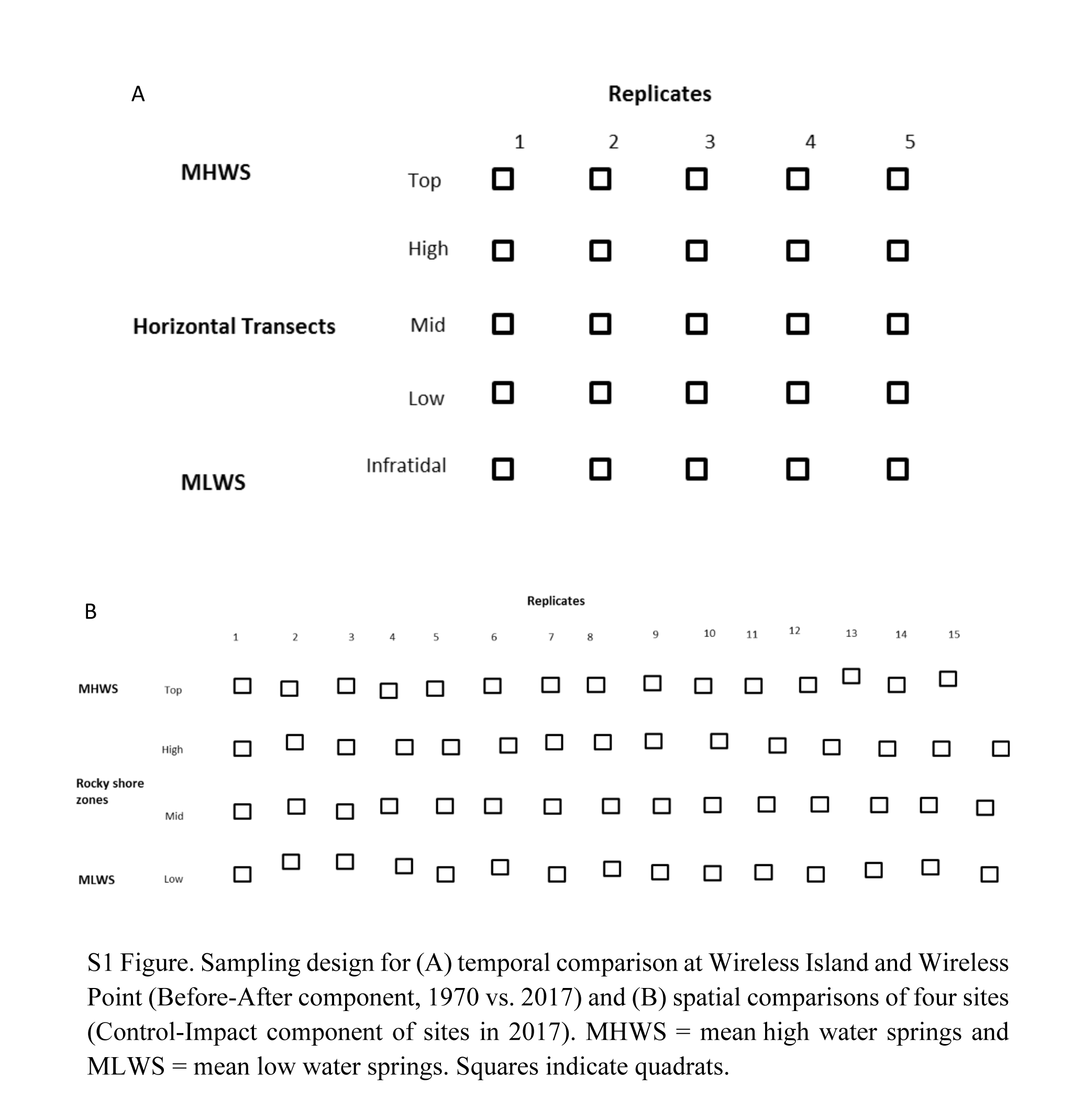

Supplement: S1 Fig — Sampling design for (A) temporal comparison at Wireless Island and Wireless Point (Before-After component, 1970 vs. 2017) and (B) spatial comparisons of four sites (Control-Impact component of sites in 2017). MHWS = mean high water springs and MLWS = mean low water springs. Squares indicate quadrats. (TIF) [file pone.0294404.s001.tif]

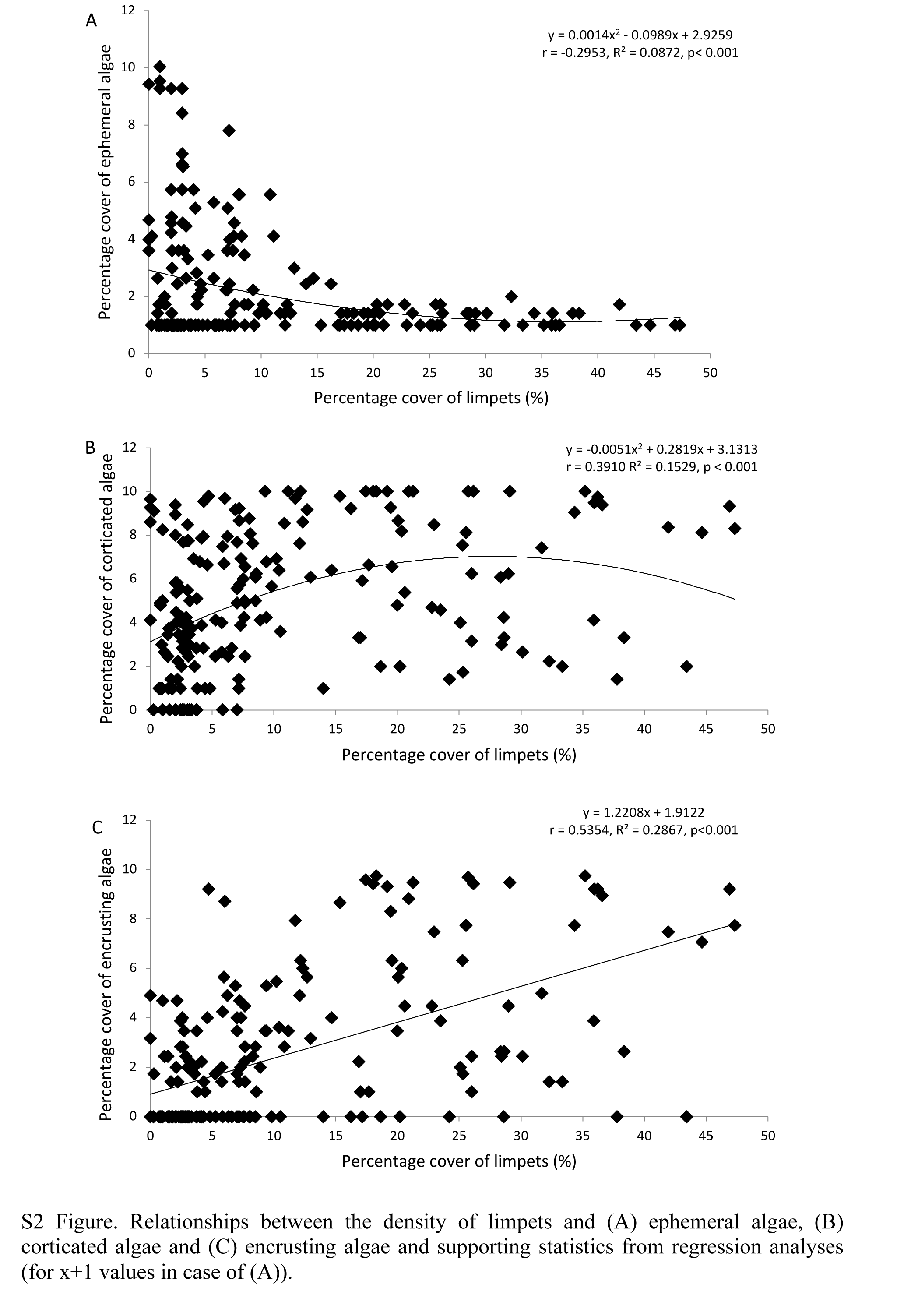

Supplement: S2 Fig — Relationships between the density of limpets and (A) ephemeral algae, (B) corticated algae and (C) encrusting algae and supporting statistics from regression analyses (for x+1 values in case of (A)). (TIF) [file pone.0294404.s002.tif]
