# Supplementary material for: Effects of harvesting and an invasive mussel on intertidal rocky shore communities based on historical and spatial comparisons
Source: PLoS One. 2024 Feb 8;19(2):e0294404. doi: 10.1371/journal.pone.0294404 (PMC10852263; doi:10.1371/journal.pone.0294404)
Supplement: S1 Table — Asterisks indicate significant effects. (DOCX) [file pone.0294404.s003.docx]

**S1 Table.** Two-way ANOVAs of the densities of *C. granatina* and *S. granularis* comparing temporal differences with factors Site and Time, and their interaction. Asterisks indicate significant effects.

| **Source** | **Df** | **SS** | | **MS** | | **Pseudo-F** | | **P(perm)** | |
| --- | --- | --- | --- | --- | --- | --- | --- | --- | --- |
| *C. granatina* | | | | | | | | | |
| Site | 1 | 10.36 | 10.36 | | 8.79 | | <0.01* | |  |
| Year | 1 | 64.76 | 64.76 | | 54.94 | | <0.01* | |  |
| Site × Year | 1 | 11.70 | 11.70 | | 9.92 | | 0.01* | |  |
| Residuals | 90 | 106.09 | 1.18 | |  | |  | |  |
| *S. granularis* | | | | | | | | |  |
| Site | 1 | 19.50 | 19.50 | | 11.42 | | <0.01* | |  |
| Year | 1 | 0.12 | 0.12 | | 0.07 | | 0.80 | |  |
| Site × Year | 1 | 0.33 | 0.33 | | 0.19 | | 0.66 | |  |
| Residuals | 90 | 153.63 | 1.71 | |  | | |  | |
