# Supplementary material for: Effects of harvesting and an invasive mussel on intertidal rocky shore communities based on historical and spatial comparisons
Source: PLoS One. 2024 Feb 8;19(2):e0294404. doi: 10.1371/journal.pone.0294404 (PMC10852263; doi:10.1371/journal.pone.0294404)
Supplement: S2 Table — Asterisks indicate significant effects. (DOCX) [file pone.0294404.s004.docx]

**S2 Table.** Results of two-way nested ANOVAs of the densities of *C. granatina*, *S. argenvillei* and *S. granularis* with factors protection level and site (nested in protection level). Asterisks indicate significant effects.

| **Source** | **Df** | **SS** | **MS** | **Pseudo-F** | | | **P(perm)** | |
| --- | --- | --- | --- | --- | --- | --- | --- | --- |
| *C. granatina* | | | | | | | | |
| Protection level | 1 | 31.80 | 31.84 | | 9.39 | <0.01* | |  |
| Site (Protection level) | 2 | 39.90 | 13.30 | | 3.92 | <0.01* | |  |
| Residuals | 172 | 583.01 | 3.39 | |  |  | |  |
| *S. argenvillei* | | | | | | | |  |
| Protection level | 1 | 75.11 | 75.11 | | 54.03 | <0.01* | |  |
| Site (Protection level) | 2 | 90.85 | 45.42 | | 32.67 | <0.01* | |  |
| Residuals | 56 | 77.86 | 1.39 | |  | |  | |
| *S. granularis* | | | | | | | | |
| Protection level | 1 | 12.80 | 12.80 | | 3.86 | | 0.05 | |
| Site (Protection level) | 2 | 23.40 | 11.71 | | 3.53 | | 0.03* | |
| Residuals | 230 | 763.20 | 3.32 | |  | |  | |
