# Supplementary material for: Effects of harvesting and an invasive mussel on intertidal rocky shore communities based on historical and spatial comparisons
Source: PLoS One. 2024 Feb 8;19(2):e0294404. doi: 10.1371/journal.pone.0294404 (PMC10852263; doi:10.1371/journal.pone.0294404)
Supplement: S3 Table — Asterisks indicate significant effects. (DOCX) [file pone.0294404.s005.docx]

**S3 Table.** Results of two-way nested ANOVAs of the sizes (shell lengths) of *C. granatina*, *S. argenvillei* and *S. granularis* with factors protection level and site nested in protection level. Asterisks indicate significant effects.

| **Source** | **Df** | **SS** | **MS** | **Pseudo-F** | | **P(perm)** | |
| --- | --- | --- | --- | --- | --- | --- | --- |
| *C. granatina* | | | | | | | |
| Protection level | 1 | 41.50 | 41.50 | | 20.13 | <0.01* |  |
| Site (Protection level) | 2 | 23.60 | 11.80 | | 5.72 | <0.01* |  |
| Residuals | 209 | 430.90 | 2.06 | |  |  |  |
| *S. argenvillei* | | | | | | |  |
| Protection level | 1 | 79.76 | 79.76 | | 59.57 | <0.01* |  |
| Site (Protection level) | 2 | 61.45 | 30.73 | | 22.95 | <0.01* |  |
| Residuals | 218 | 291.87 | 1.34 | |  |  | |
| *S. granularis* | | | | | | | |
| Protection level | 1 | 3.10 | 3.13 | | 2.58 | 0.11 | |
| Site (Protection level) | 2 | 132.40 | 66.21 | | 54.45 | <0.01* | |
| Residuals | 291 | 353.90 | 1.22 | |  |  | |
