# Supplementary material for: Effects of harvesting and an invasive mussel on intertidal rocky shore communities based on historical and spatial comparisons
Source: PLoS One. 2024 Feb 8;19(2):e0294404. doi: 10.1371/journal.pone.0294404 (PMC10852263; doi:10.1371/journal.pone.0294404)
Supplement: S4 Table — Asterisks indicate significant effects. (DOCX) [file pone.0294404.s006.docx]

**S4 Table**. Two-way ANOVA of the densities and sizes of *Scutellastra* *granularis* with factors year and zones, and their interaction. Asterisks indicate significant effects.

| **Source** | | **Df** | | **SS** | **MS** | | **F-value** | | **P-value** |
| --- | --- | --- | --- | --- | --- | --- | --- | --- | --- |
| **Density** | | | | | | | | | |
| Year | 1 | | 0.25 | | 0.25 | 0.47 | | 0.50 | |
| Zone | 4 | | 18.19 | | 4.55 | 8.43 | | <0.01* | |
| Year × Zone | 4 | | 54.16 | | 13.54 | 25.11 | | <0.01* | |
| Residuals | 42 | | 22.65 | | 0.54 |  | |  | |
| **Size** |  | |  | |  |  | |  | |
| Year | 1 | | 23.75 | | 23.75 | 56.45 | | <0.01* | |
| Zone | 4 | | 114.85 | | 28.71 | 68.25 | | <0.01* | |
| Year × zone | 4 | | 40.87 | | 10.22 | 24.29 | | <0.01* | |
| Residuals | 696 | | 292.78 | | 0.42 |  | | |  |
