# Supplementary material for: Effects of harvesting and an invasive mussel on intertidal rocky shore communities based on historical and spatial comparisons
Source: PLoS One. 2024 Feb 8;19(2):e0294404. doi: 10.1371/journal.pone.0294404 (PMC10852263; doi:10.1371/journal.pone.0294404)
Supplement: S5 Table — Asterisks indicate significant effects. (DOCX) [file pone.0294404.s007.docx]

**S5 Table.** Multivariate generalized linear model analysis of the community composition to test for differences between years and among intertidal zones, and their interaction. Asterisks indicate significant effects.

| **Wireless Island** | | | | | | **Wireless Point** | | | |
| --- | --- | --- | --- | --- | --- | --- | --- | --- | --- |
| **Source** | **Res. Df** | | **Df. Diff** | **Dev** | **Pr (>Dev)** | **Res. Df** | **Df. Diff** | **Dev** | **Pr (>Dev)** |
| Intercept | | 48 |  |  |  | 37 |  |  |  |
| Year | | 47 | 1 | 118.20 | 0.001* | 36 | 1 | 67.47 | 0.001* |
| Zone | | 43 | 4 | 129.10 | 0.001* | 33 | 3 | 218.55 | 0.001* |
| Year × Zone | | 39 | 4 | 121.90 | 0.001* | 30 | 3 | 47.38 | 0.003* |
