# Supplementary material for: Effects of harvesting and an invasive mussel on intertidal rocky shore communities based on historical and spatial comparisons
Source: PLoS One. 2024 Feb 8;19(2):e0294404. doi: 10.1371/journal.pone.0294404 (PMC10852263; doi:10.1371/journal.pone.0294404)
Supplement: S6 Table — P-values are adjusted for multiple testing using a step-down resampling algorithm (Wang et al. 2012). Goodness-of-fit of the models is indicated by the residual deviance (Dev), with lower values indicating better fit. Changes in the abundance over time are shown as increases (+), decreases (-) or no change (0). Blank cells = absence or scarcity that prevented analysis. (DOCX) [file pone.0294404.s008.docx]

**S6 Table.** Univariate tests for differences in abundance over time and among intertidal zones. P-values are adjusted for multiple testing using a step-down resampling algorithm (Wang et al. 2012). Goodness-of-fit of the models is indicated by the residual deviance (Dev), with lower values indicating better fit. Changes in the abundance over time are shown as increases (+), decreases (-) or no change (0). Blank cells = absence or scarcity that prevented analysis.

| **Wireless Island** | | | | | | | | **Wireless Point** | | | |
| --- | --- | --- | --- | --- | --- | --- | --- | --- | --- | --- | --- |
| **Source** | **Dev** | | **Pr (>Dev)** | | | | **Change** | **Dev** | **Pr (>Dev)** | | **Change** |
| *Mytilus galloprovincialis* | | | | | | | | | | | |
| Year | | 63.92 | | <0.01 | | | + |  |  | |  |
| Zone | | 18.55 | | 0.02 | | |  |  |  | |  |
| Year × Zone | | 0.00 | | 0.79 | | |  |  |  | |  |
| *Aulacomya atra* | | | | | | | | | | | |
| Year | | 0.04 | | 0.96 | | | 0 | 2.83 | 0.19 | |  |
| Zone | | 3.23 | | 0.33 | | |  | 11.65 | 0.02 | |  |
| Year × Zone | | 14.00 | | 0.10 | | |  | 10.65 | 0.04 | | – |
| *Cymbula granatina* | | | | | | | | | | | |
| Year | | 9.52 | | 0.01 | | | – | 32.01 | <0.01 | | – |
| Zone | | 16.62 | | 0.02 | | |  | 69.49 | <0.01 | |  |
| Year × Zone | | 15.51 | | 0.08 | | |  | 8.97 | 0.06 | |  |
| *Cymbula oculus* | | | | | | | | | | | |
| Year | 0.01 | | | 0.96 | | | 0 | 16.73 | <0.01 | |  |
| Zone | 28.98 | | | < 0.01 | | |  | 12.43 | 0.02 | |  |
| Year × Zone | 7.15 | | | 0.35 | | |  | 9.73 | 0.04 | | - |
| *Scutellastra barbara* | | | | | | | | | | | |
| Year | 0.22 | | | 0.89 | | | 0 |  | |  |  |
| Zone | 16.62 | | | 0.04 | | |  |  | |  |  |
| Year × Zone | 8.26 | | | 0.27 | | |  |  | |  |  |
| *Scutellastra granularis* | | | | | | | | | | | |
| Year | 2.68 | | | 0.28 | | | 0 | 0.34 | | 0.50 | 0 |
| Zone | 7.01 | | | 0.26 | | |  | 58.61 | | < 0.01 |  |
| Year × Zone | 53.77 | | | <0.01 | | |  | 2.25 | | 0.77 |  |
| Ephemeral algae | | | | | | | | | | | |
| Year | | 6.62 | | 0.05 | | | + | 11.30 | | 0.01 |  |
| Zone | | 6.41 | | 0.26 | | |  | 2.12 | | 0.48 |  |
| Year × Zone | | 4.07 | | 0.40 | | |  | 14.86 | | 0.02 | + |
| Corticated algae | | | | | | | | | | | |
| Year | | 33.56 | | <0.01 | | | + | 1.42 | | 0.24 | 0 |
| Zone | | 23.66 | | 0.01 | | |  | 35.76 | | <0.01 |  |
| Year × Zone | | 6.75 | | 0.35 | | |  | 0.33 | | 0.77 |  |
| Encrusting algae | | | | | | | | | | | |
| Year | 1.60 | | | | 0.38 | | 0 | 4.21 | | 0.10 | 0 |
| Zone | 10.50 | | | | 0.10 | |  | 23.50 | | <0.01 |  |
| Year × Zone | 12.44 | | | | 0.12 |  | | 1.237 | | 0.77 |  |
