# Supplementary material for: Effects of harvesting and an invasive mussel on intertidal rocky shore communities based on historical and spatial comparisons
Source: PLoS One. 2024 Feb 8;19(2):e0294404. doi: 10.1371/journal.pone.0294404 (PMC10852263; doi:10.1371/journal.pone.0294404)
Supplement: S7 Table — (DOCX) [file pone.0294404.s009.docx]

**S7 Table.** Two-way nested PERMANOVA with factors protection level (harvested vs. no-take) and site (nested in protection level) showing their effects on community composition in the four intertidal zones.

| **Source** | **Df** | | **SS** | | **MS** | **Pseudo-F** | **P(perm)** |
| --- | --- | --- | --- | --- | --- | --- | --- |
| **Low shore** | | | | | | | |
| Protection level | 1 | 52468 | | 52468 | | 50.414 | <0.01* |
| Site (Protection level) | 2 | 36574 | | 18287 | | 17.571 | <0.01* |
| Residuals | 56 | 58282 | | 1040.8 | |  |  |
| **Mid shore** | | | | | | | |
| Protection level | 1 | 21268 | | 21268 | | 19.578 | <0.01* |
| Site (Protection level) | 2 | 45282 | | 22641 | | 20.842 | <0.01* |
| Residuals | 58 | 63007 | | 1086.3 | |  |  |
| **High shore** | | | | | | | |
| Protection level | 1 | 18953 | | 18953 | | 17.966 | <0.01* |
| Site (Protection level) | 2 | 58875 | | 29437 | | 27.905 | <0.01* |
| Residuals | 50 | 52745 | | 1054.9 | |  |  |
| **Top shore** | | | | | | | |
| Protection level | 1 | 30500 | | 30500 | | 33.104 | <0.01* |
| Site (Protection level) | 2 | 63043 | | 31522 | | 34.213 | <0.01* |
| Residuals | 52 | 47910 | | 921.35 | |  |  |
